# Dual First and Second Surface Solar Mirrors of Polished WS_2_ and Silver by Dynamical Chemical Plating Technique on Polycarbonate

**DOI:** 10.3390/polym16131951

**Published:** 2024-07-08

**Authors:** Coraquetzali Magdaleno López, José de Jesús Pérez Bueno, Alejandra Xochitl Maldonado Pérez, Yunny Meas Vong, Jorge Morales Hernández, José Emanuel Ambrosio Juárez, Iván Toledo Manuel, José Antonio Cabello Mendez, David Meneses Rodríguez

**Affiliations:** 1 Instituto Tecnológico Superior de Abasolo. Blvd. Cuitzeo de los Naranjos #401, Col. Cuitzeo de los Naranjos, Abasolo C.P. 36976, Guanajuato, Mexico; 2 Centro de Investigación y Desarrollo Tecnológico en Electroquímica, S.C., Pedro Escobedo, Querétaro C.P. 76703, Querétaro, Mexico; amaldonado@cideteq.mx (A.X.M.P.); yunnymeas@cideteq.mx (Y.M.V.); jmorales@cideteq.mx (J.M.H.); 3 Instituto Mexicano del Transporte, Carretera El Colorado—Galindo Km. 12 Col. Sanfandila, Pedro Escobedo, Querétaro C.P. 76703, Querétaro, Mexico; antonio.cabello@imt.mx; 4 Cátedras—Centro de Investigación y de Estudios Avanzados Unidad Mérida, Km. 6 Antigua Carretera a Progreso Apdo. Postal 73, Cordemex, Mérida C.P. 97310, Yucatán, Mexico

**Keywords:** dynamic chemical plating (DCP), tungsten disulfide (WS_2_), flame treatment, mechanical exfoliation method

## Abstract

This work proposes for the first time protecting–reflecting on both sides of plated mirrors and a solution to polycarbonate surface vulnerability to weathering and scratching using tungsten disulfide (WS_2_) by mechanical polishing. The ability of the dynamic chemical plating (DCP) technique to deposit Ag films at the nanometer scale on a polycarbonate (PC) substrate and its characteristics to be metallized is also shown. These deposits hold significant promise for concentrated solar power (CSP) applications. Complementarily, the application of WS_2_ as a reflective film for CSP by mechanical polishing on smooth polycarbonate surfaces is both novel and practical. This technique is innovative and scalable without needing reactants or electrical potential, making it highly applicable in real-world scenarios, including, potentially, on-site maintenance. The effects of surface morphology and adhesion, and the reflectivity parameters of the silver metallic surfaces were investigated. Wettability was investigated because it is important for polymeric surfaces in the activation and metal deposition immediately after redox reactions. The flame technique improved wettability by modifying the surface with carbonyl and carboxyl functional groups, with PC among the few industrial polymers that resisted such a part of the process. The change in the chemical composition, roughness, and wettability of the surfaces effectively improved the adhesion between the Ag film and the PC substrate. However, it did not significantly affect the adhesion between PC and WS_2_ and showed its possible implementation as a first surface mirror. Overall, this work provides a scalable, innovative method for improving the durability and reflectivity of polycarbonate-based mirrors, with significant implications for CSP applications.

## 1. Introduction

Among the various renewable energy generation methods, concentrated solar power (CSP) has gained significant interest due to its features, such as improved energy efficiency and the ability to collect solar thermal energy at low temperatures [1,2,3]. However, solar power plants are only profitable if the service life of their components, particularly reflective mirrors, exceeds 20 years [4,5].

Solar reflectors are one of the main components of CSP devices [6]. Any imperfections in these reflectors impact their optical performance, affecting the efficiency of the plant. This issue has prompted recent research on manufacturing processes [1], durability [7], and the degradation of reflective and protective layers [8].

Silver glass reflectors are commonly used in CSP systems [9] among the different types of solar mirrors. They are classified as second-surface reflectors based on a thin layer of reflective silver protected by a 1 to 4 mm glass substrate on the front and, typically, several layers of protective paint on the back [10,11]. In contrast, there are first-surface mirrors, such as those used in telescopes [12,13]. These systems have gained popularity because they do not require high-purity glass as a substrate [7,14,15].

The performance of mirrors depends primarily on the films deposited on the substrates [16,17]. Silver (Ag), which has a hemispherical reflectivity of 95–99% in the visible region and 90–95% in the near-infrared, is known to be one of the best solar reflectors [18,19], along with aluminum at 85–90% [20,21,22]. The spectral hemispherical reflectance calculated with the Fresnel equations depends on the complex refractive index, wavelength, incidence angle, roughness, absorption coefficient, and electrical conductivity. Various methods have been used to deposit silver films on substrates, including magnetron sputtering [23,24], thermal evaporation [25], electrodeposition [26], and non-electrolytic coating [27,28].

A suitable way to apply reflective coatings on non-conductive substrates, such as glass or plastics, is through an electroless plating process [29,30]. This process consists of the spontaneous reaction of different solutions without applying an electric current. However, the surfaces are immersed in a bath, requiring large amounts of solution.

Another process for applying thin metallic films to non-conductive substrates is dynamic chemical plating (DCP). It is a wet chemical deposition technique [31,32,33]. The novelty of DCP is based on a highly controlled sequential supply of materials (metal ions, such as Ni^2+^, Cu^2+^, Co^2+^, or Ag^+^) and an electron source (reducing agent) on the substrate.

DCP is a modified non-electrolytic deposition process in which a chemical reaction occurs without applied current and can coat irregular or non-conductive surfaces. A double-nozzle gun simultaneously sprays two different solutions containing the precursors onto the surface of the substrate [34,35]. After the projection and mixing of the two independent liquid jets on the surface, at a pressure of about 500 kPa (5 bar), the precursors react spontaneously through redox reactions, forming a thin, thermodynamically unstable liquid film on the substrate surface. DCP has enabled the production of compact, dense, and adherent metal coatings, e.g., with Ag and Cu or Ni–B [34], on substrates at room temperature, with deposition rates around 10 m/h.

On the other hand, tungsten disulfide (WS_2_) has been widely used for different applications, such as sensors [36], catalysts for hydrocracking oil [37], protecting polymers [38], and textiles for oil absorption or oil/water separation [39].

WS_2_ has been used for saturable absorber mirrors with high-damage-resistant characteristics for solid-state lasers such as Q-switched fiber lasers [40,41]. Han et al. [42] made a plasmonic nanocavity with a nanoparticle on a mirror system composed of Ag nanocubes on an Ag film separated from an Al_2_O_3_ spacer. This was coupled with a monolayer of WS_2_ for studying a second-harmonic generator. On the other hand, Huang et al. [43] studied a hybrid nanocavity composed of a silicon nanoparticle and a gold film. Again, this was coupled with a monolayer of WS_2_ for studying a second-harmonic generator.

WS_2_ has another important application as a low-friction dry lubricant coating, competing with or complementing MoS_2_ [38]. Thus, it can reduce friction and noise. Products such as Dicronite^®^ and DL-5^®^, in accordance with the standards AMS2530 [44], provide the lubricity effect on surfaces by applying a coating.

This work aims to develop a dynamic solar reflector of Ag and WS_2_ on a highly reflective polycarbonate substrate, which has a potential application as a concentrating solar technology. The mirrors are a key element that can contribute to solve the main problem that CSP systems are addressing, which is collecting solar energy, but the costs of construction, operation, and maintenance of the facilities severely restrict them. Here, WS_2_/PC/Ag/epoxy paint is a mirror protecting–reflecting on both sides, directed to a collecting point. The WS_2_ acts as a thin reflective film, complementing the reflective characteristics of silver surfaces while also protecting the front side of the PC, which can be directly restored on-site. Here, the morphological and optical characteristics of the deposits are presented.

## 2. Materials and Methods

### 2.1. Silver Metallization of PC Samples

Metallic silver was deposited by DCP on solid polycarbonate sheets (LEXANTM 9043, SABIC (Saudi Basic Industries Corporation), Riyadh, Saudi Arabia) of 1.22 × 1.22 cm and 1.5 mm thick. Tests were conducted with different thicknesses of the metal coating, from about 45 to 245 nm. This electroless technique allowed the mirror finish to be produced under ambient conditions by sequential spraying, as the aerosols were projected onto the surface of the substrate.

**Figure 1:**
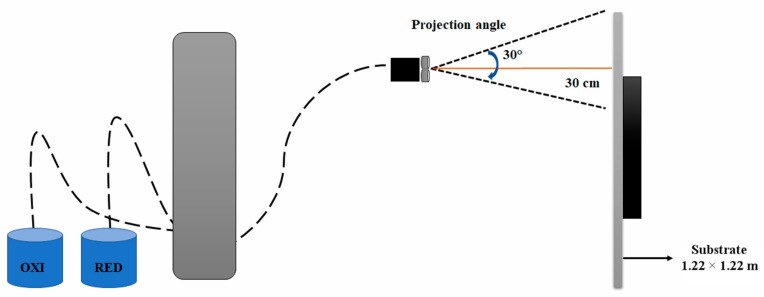
Schematic representation of dynamic chemical plating (DCP) apparatus.

The DCP technique consists of the controlled reduction of a metal cation [31,35]. Two solutions were prepared, the first containing the salts of the metal to be deposited and the second the electron reserve in the form of a reducing chemical compound. With the help of a device (aerosol), the aqueous solutions were projected simultaneously and in the same proportion on the substrate in the form of tiny droplets (Figure 1). Mixing the two aqueous solutions causes a redox reaction on the surface of the substrate (Equation (1)).

(1) $$M_{solution}^{n+}+{Red}_{solution}\to M_{surface}+{Ox}_{solution}^{n+}$$

The coalescence of the droplets forms a liquid film that covers and wets the surface exposed to the projection, obtaining a homogeneous and continuous metallic deposit.

**Figure 2:**
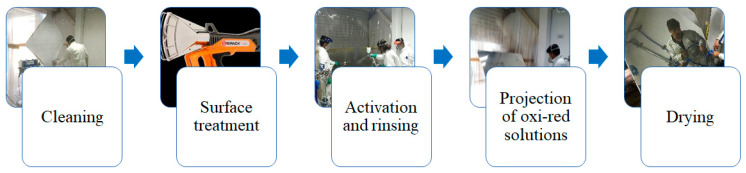
Diagram of the DCP metallization process.

The DCP method consists of five stages: cleaning, surface treatment, activation and rinsing, spraying of the solutions, and rinsing–drying (Figure 2).

### 2.2. Surface Treatment of PC Samples

Improved coating adhesion behavior is necessary due to the poor adhesion properties inherent to polycarbonate, attributed to its low surface free energy and lack of polar groups. The purpose of the surface treatment is to insert functional groups to improve wettability and adhesion to the metal film, so a flame treatment was applied to the surface. The flame treatment was conducted with a Ripack 3000 heat gun (Ripack, IPS Group; Muret, France). The parameters used for the treatment technique are shown in Table 1. The flame was applied manually on the PC plates, first with horizontal movements and later with vertical displacement. The time took about 2.5 s, from one side to the other, for the 1.23 m wide PC plates. The procedure was repeated three times to ensure complete wettability of the entire surface with a pause between them to avoid overheating of the PC.

### 2.3. Coating of WS_2_ by Polishing

The WS_2_ coatings were applied using a mechanical polishing technique with Truper^®^ 15399 LIRO-5N and 102633 PULA-6AM polishers (Truper^®^; Jilotepec, State of Mexico, Mexico). Tests include samples of WS_2_ coatings on PCs with and without flame treatment. The WS_2_ particles were spread on the PC without its protective film. The powder was spread for about 15 min using the polishing machine. Then, it was washed with neutral soap, rinsed with water to remove the excess, and dried.

**Figure 3:**
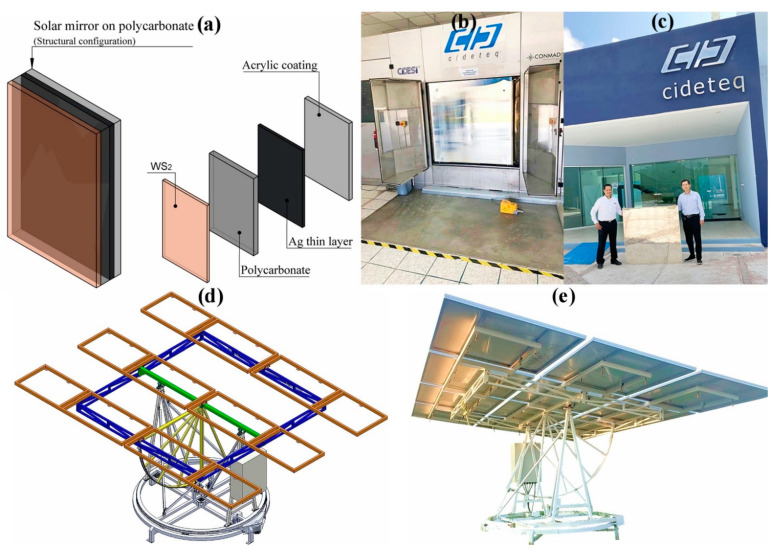
(**a**) Solar mirror configuration on polycarbonate showing the mirror layers: WS_2_, 1.3 mm thick PC plate, nanometric reflective silver coating deposited by DCP, and acrylic paint protective coating. (**b**) Automated facilities for silver DCP mirrors. (**c**) Silver mirror piece. (**d**) Heliostat model. (**e**) Heliostat constructed and installed with twelve silver mirrors of polycarbonate.

Figure 3a shows the proposed mirror configuration with nanometric reflective silver surfaces on the back of the PC plates, complemented with WS_2_ on the front side. The 1.3 mm thick PC plates are proposed as substrates, and an acrylic coating was deposited on the silver to protect it from the weather conditions.

Figure 3b shows a finished silver mirror, and Figure 3c shows an individual mirror of silver on PC with an aluminum frame. Figure 3d shows the designed heliostat structure to hold twelve framed mirrors, and Figure 3e shows the constructed and installed structure, which wears the twelve silver mirrors on PC pieces.

### 2.4. Characterization

The morphology of the surface was determined by digital light microscopy using a Keyence VHX-5000 microscope (Keyence; Osaka, Japan) with a 5000× optical objective, model VH-Z500R/Z500T (Keyence; Osaka, Japan), with a numerical aperture NA = N sin θ = 0.82 (N refractive index around the objective/N~1 for air).

The chemical state of silver was analyzed by X-ray photoelectron spectroscopy (XPS) using a Thermo Scientific™ K-Alpha XPS spectrometer (Thermo Fisher Scientific; Waltham, Massachusetts, USA) using internal pressure in the analysis chamber of 6.5 × 10^−9^ mbar and a hemispherical analyzer with dual 180° focus and a 128-channel detector. The spectrometer used a monochromatic Al Kα X-ray source (hν = 1486.68 eV) and an analysis radius of 400 μm and an ion gun with an energy range of 100 to 4000 eV. The acquired high-resolution Ag 3d spectrum was processed with Avantage software (Thermo Fisher Scientific. (2024). version 6.7. Waltham, MA, USA), referencing the C1s bond of adventitious carbon at 284.8 eV [29].

Adequate adhesion between metallic or protective coatings and substrates was successfully obtained. The adhesion properties of the surfaces were evaluated using Scotch tape adhesion tests. Adhesion classification was performed using the cross-cut tape test method (Precision Gage & Tool Co.; Dayton, OH, USA) in accordance with ASTM D 3359-23 [45]. The tests were conducted at room temperature. Three repetitions were performed and averaged to obtain adhesion values.

The contact angle test measured the surface wettability behavior. Contact angle and surface energy measurements were made using the sessile drop technique with a KRÜSS device (Hamburg, Germany), model DSA30, at a room temperature of 20–25 °C. A drop of deionized water (10 μL droplets) was applied to the surface, the range of the sessile drop was from 0 to 180°, and the hydrophobic or hydrophilic character of the analyzed surface was determined. The model used for the measurement was the Young–Laplace.

## 3. Results and Discussion

### 3.1. Effect of Flame Activation on PC Surface Behavior

A pre-treatment was performed on the surface of the polycarbonate to improve the adhesion between the silver layers. The corresponding chemical and topographic changes on the surface of the flame-treated samples were characterized and linked to adhesion properties. It is also important to note that, despite the relatively high temperatures, the short duration of the treatment allows the activation of polymers without damaging the surface visually.

**Figure 4:**
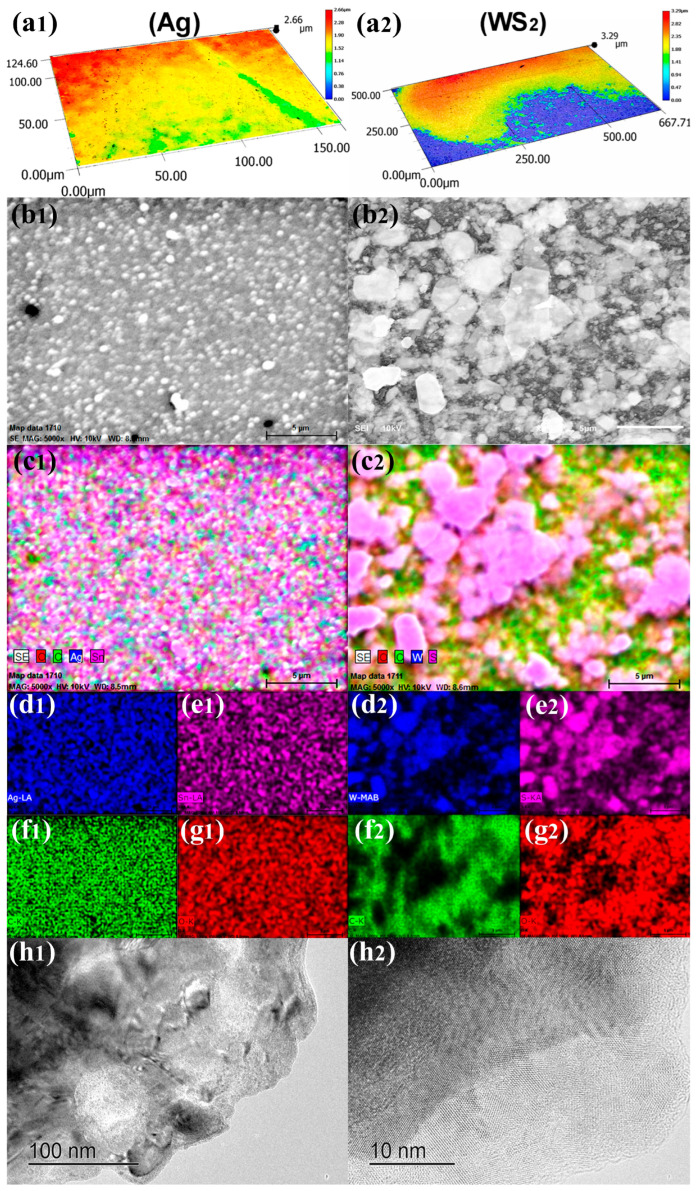
Topographic and chemical characterization of silver (1st column) and WS_2_ (2nd column) coatings on polycarbonate. (**a**) Optical micrograph; (**b**) SEM micrograph at 5000×; (**c**) EDS mapping showing the global distribution of elements; (**d**) shows Ag or W; (**e**) shows Sn or S; (**f**) shows C; (**g**) shows O; (**h**) show HR-TEM micrographs of WS_2_ with a scale bar of 100 nm and 10 nm, respectively.

The topography of the surface strongly influences adhesion properties. The decisive factors are the size of the contact area and the size of the voids between the adherents. Figure 4 shows the topographic images of the silver and WS_2_ coatings. The silver coatings, made of nucleation of atoms by reducing silver ions, were more homogeneous and flat than the overlapping lamellar particles of WS_2_ (Figure 4a,b). The scales show a surface with more waviness for the WS_2_, with some irregularities on and within the layer attributed to the polishing process, which, despite being mechanically applied, was homogeneous and reflected light specularly instead of diffusely.

These surface topography changes did not result from thermal effects or chemical reactions of the flame species with the surface, such as carbonyl and carboxyl groups [46]. Physically, these changes are not significant (there is no wear or degradation), but the chemical changes were responsible for surface energy, contact angle, and adhesion variations.

Figure 4c of EDS mapping for Ag and WS_2_ shows a marked difference on the surfaces. The WS_2_ surface has different particles contrasting with the green carbon signal from the substrate underneath (polycarbonate) but which is not directly exposed. In the center of SEM figures for WS_2_, there is a tiny flat particle of about 5 mm that is semitransparent for electrons. Figure 4d–g show the individual mapping for each element in, both cases Ag and WS_2_. Figure 4h,i show the micrographs of high-resolution transmission electron microscopy (HR-TEM) for WS_2_ with scale bars of 100 nm and 10 nm. The lamellar particles were highly crystalline and Moiré patterns were common because of the displacement between consecutive lamellas.

**Figure 5:**
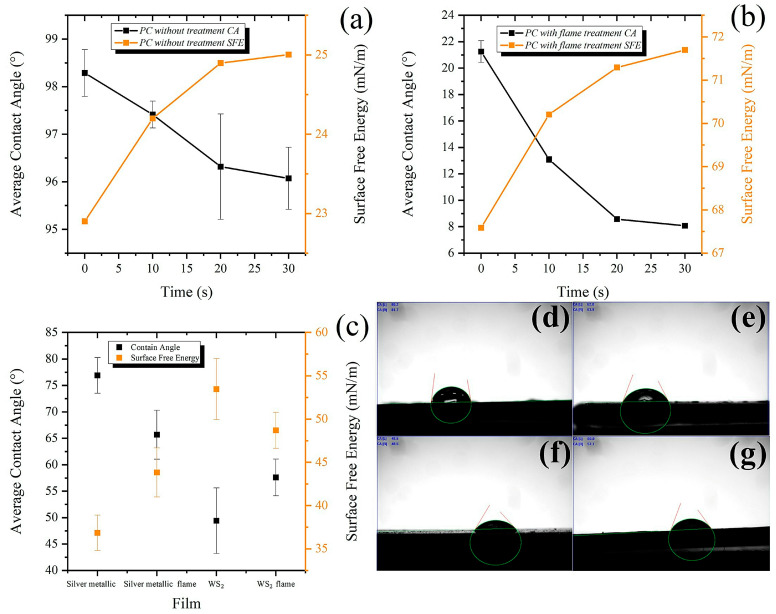
Contact angle and surface free energy measurements of (**a**) surfaces without flame treatment, (**b**) surfaces with flame treatment, and (**c**) a comparison between silver and WS_2_ surfaces without and with flame treatment. Examples of contact angle images for the surfaces of (**d**) silver, (**e**) flame-treated silver, (**f**) WS_2_, and (**g**) flame-treated WS_2_.

The wettability of a substrate’s surface is usually related to the quality of adhesion that can be achieved after coating it with another material [47]. Figure 5 shows the variation of contact angle values and surface free energy measured on polycarbonate surfaces before and after flame treatment using a deionized water droplet, where the contact angle values of the non-flame-treated surface are higher than those of the treated surface.

Figure 5c compares the different coatings and the effects of the flame treatment. The untreated surface had low wettability in the different analysis times, i.e., the surface of the CP is hydrophobic, with an initial value of 98.3° ± 0.27°. However, after heat treatment, the contact angle values tended to decrease, so it was observed that the surface was more hydrophilic, where the contact angle value at time zero was 21.4° ± 0.4°. The flame treatment significantly influenced the hydrophilic behavior of all the tested surfaces, causing increased wetting, which led to a decrease in the contact angle and an increase in the free energy of the surface.

The decay rate (x) is calculated between the minimum and maximum contact angles (*CA_min_* and *CA_max_*, respectively) [48], as shown in Equation (2).

(2) $$x=100\left(1-\frac{{CA}_{min}}{{CA}_{max}}\right)$$

The obtained contact angle values demonstrate excellent wettability of the treated surface, which induces adequate adhesion between the substrate and the coating. This improvement in wetting behavior is caused by the formation of functional groups on the surface of the polymer.

The decrease in the contact angle with the activation of the flame treatment implies an increase in the surface free energy of the polycarbonate and, therefore, an increase in the adhesion between the surface and the water droplet, i.e., a higher amount of energy per unit area is required to separate them [49].

Contrary to the expected effect, coated surfaces that were previously flame-treated had higher contact angles than untreated surfaces. Usually, flame treatment and corona electrical discharges or plasma improve wettability by lowering the contact angle [48]. This result was the case for preparing the PC surfaces before wetting, activating, and silver deposition.

On the other hand, comparing the silver and WS_2_ surfaces revealed a higher surface energy for the silver coatings, which can be attributed to the hydrophobic properties of WS_2_ [50]. Higher surface energy is typically associated with higher adherence because it promotes interaction with other substances or materials. On the contrary, lower surface energy has a lower affinity with other substances or materials, which repels them and reduces adhesion.

According to the Gibbs free energy equation, increasing the surface free energy of polymers promotes condensation and wettability. It is necessary to increase the Gibbs free energy to improve the wettability area of the liquid droplet on a solid surface since the surface tension is constant and this is an intrinsic property of the material [51,52]. Therefore, the increase in PC free energy observed by decreasing the contact angle with water can promote higher homogeneity of the silver film on the PC substrate.

The polymer base of solar concentrators are substrates with a large surface area. Conventional plating techniques can be very expensive or impractical for large areas. That is why a method is required that allows a metal deposit to be made for large areas practically and efficiently in a short time. The DCP technique makes it possible to make silver deposits with the physicochemical characteristics desired for solar concentrators.

For this metallization stage, two solutions are designed: an oxidizer and reducer. The oxidizing solution consists of silver nitrate complexed by ammonia [53]. As for the reducing solution, it is made only from sugar (glucose) [54]. The reaction takes place as follows [53]:

(3) $$2{Ag}^{+}+C_{6}H_{12}O_{6}+H_{2}O\to 2Ag+{C_{6}H_{11}O_{7}}^{-}+3H^{+}$$

It is necessary to meet some basic requirements to achieve an excellent metallic layer: the one-to-one stoichiometry between the reducing and oxidizing solution and the thickness of the mixture film (metal salt and reducing solution) on the surface of the substrate. Tests were conducted with different thicknesses to study the influence of the thickness of the metal layer on optimizing the ideal parameters.

**Figure 6:**
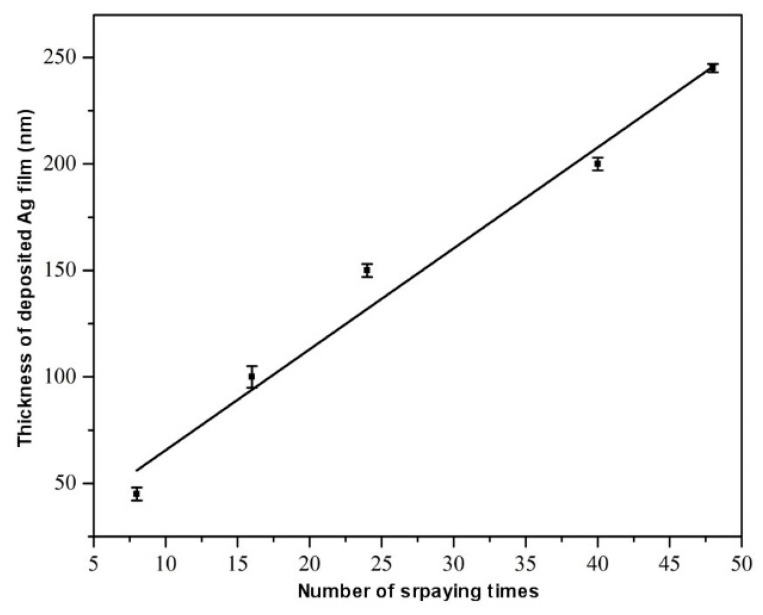
Thickness curve of the silver metallic film using the DCP technique.

Figure 6 shows the deposition thickness of the metallic silver film on the flame-treated polycarbonate substrate. The kinetic growth of the film is linear, corresponding to the increase in the number of layers. Table 2 shows the values of the number of layers placed and the thickness obtained in each, which is increasing.

However, too much thickness caused too much stress, which negatively influenced the adhesion of the film between layers, which is negative for reflectance. Therefore, sixteen spray sequences were chosen as the optimal ones for studying silver films. After sixteen spray sequences, the metal film behaved uniformly and continuously throughout the substrate.

For not exceeding the thickness of the film, the projection was kept in motion throughout the sample, i.e., from left to right and from top to bottom of the substrate. This condition allowed the reaction to occur while projecting onto different points on the polymer’s surface.

According to the results obtained for the continuous projection mode, a silver deposit can be obtained under this condition since the reaction is fast enough to eliminate the relaxation time. A continuous mode can be used to design solutions for an industrial application.

One of the most relevant properties of these surfaces is their optical behavior. Once the deposit was made, the percentage of transmittance was analyzed, and the optimal conditions for the required objectives were determined.

**Figure 7:**
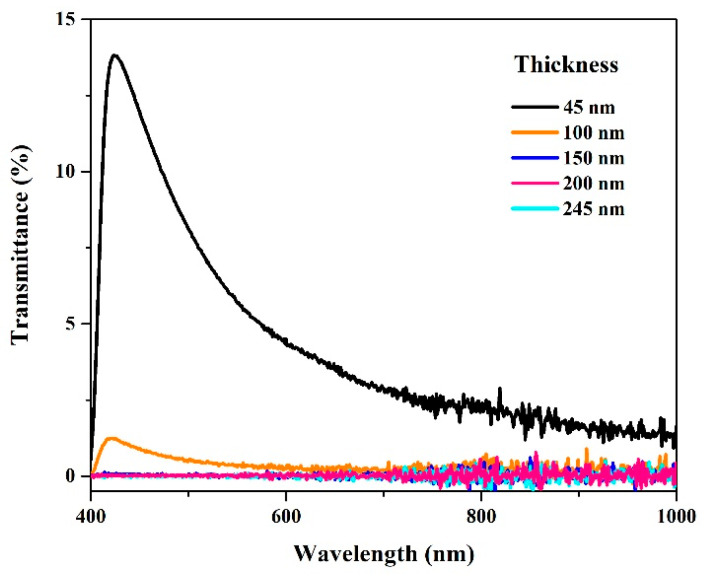
Vis-NIR spectra of the percentage of transmittance concerning the thickness of the silver metallic layer on the surface of the polycarbonate.

Figure 7 shows the spectra of the transmittance percentage compared to the thickness of the metal layer. There is a loss of 1% of transmittance in the visible region with a thickness of 100 nm. However, in the 200–400 nm region, an absorption signal is not possible because the PC substrate absorbs at this wavelength.

**Figure 8:**
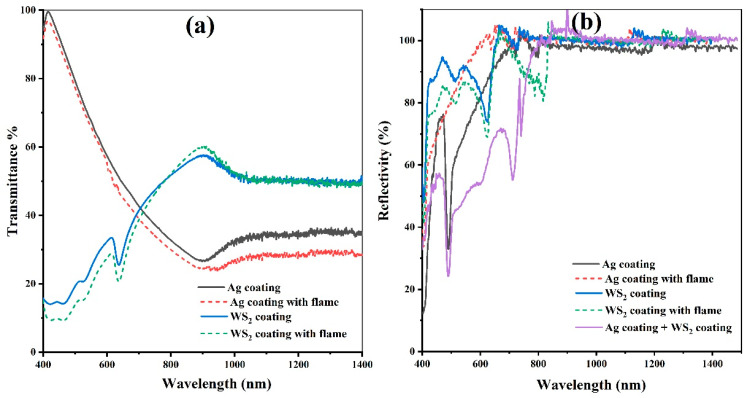
(**a**) Transmittance of the silver and WS_2_ surfaces. (**b**) Reflectance curves of the silver metal surface and WS_2_.

A percentage reflectivity analysis was performed on the approximately 100 nm silver film. The total reflectivity curve is shown in Figure 8. The silver film has high reflectivity behavior in the visible region, absorbing more radiation from the ultraviolet region. The reflectivity in the visible range is about 95%.

Figure 8 compares the transmittance (Figure 8a) and reflectance (Figure 8b) of the surfaces. Considering 100% transmittance without coating, a higher transmittance percentage can be observed in the near-UV region for the silver coating than for the WS_2_ surfaces. Figure 8a shows that the effect of the flame treatment on both types of coatings was a slight reduction in intensity, maintaining a similar spectrum shape.

Silver surface reflectance shows a significant increase in the visible region with flame treatment, attributed to eliminating residues of silver compounds that could be photoactive, darken the surface, or even cause uneven areas.

On the other hand, comparing the WS_2_ surface reflectance with and without flame treatment revealed a slight reduction in the Vis-NIR regions, which was attributed to surface lamellae orientation changes.

The Ag + WS_2_ is darker than Ag or other cases in the 400–800 nm region, but we are highlighting the specularity of the WS_2_ coating and the beneficial contribution of such a layer as protection for PC. It can keep the surface clean after rain and is a solid lubricant, like MoS_2_ or graphite, that can avoid scratches, which is a main issue for PC use as a mirror substrate.

### 3.2. Adhesion Testing

The effectiveness of coatings is primarily based on the interfacial adhesion strength between the coatings and the substrate surface. Since adhesion is one of the most important factors in the coating application, pretreatment of the substrate is needed to improve its adhesion to the film [31]. Such pretreatment involves decontamination, sensitization, and activation of the substrate to aid the subsequent formation of the metal layer [33].

The adhesion between the silver film and the substrate was tested using Scotch tape, a suitable method for laboratory-scale preparation. A 5 × 5 mm grid pattern was made on the metal film by cross-section with a distance of 1 mm between lines. A 2.5 inch piece of tape (3M Scotch^®^ tape) was applied over the cut and manually removed as quickly as possible. Adherence was assessed semi-quantitatively by comparison and description in the same set. Table 3 shows the results obtained from the metallization adhesion test for each layer placed on the substrate. The codes in this table show the percentage of the area removed from a substrate according to ASTM D-3359-23 [45]. The adhesion scale ranges from 5B to 0B.

The tests showed a higher percentage of the detached area after depositing 24 layers, increasing the percentage with 48 layers to more than 65%.

Table 3 data show that as the layers added to the substrate increased, the percentage of the removed area increased. This condition is attributed to the fact that, as the thickness increases, the coating becomes brittle and begins to fracture.

**Figure 9:**
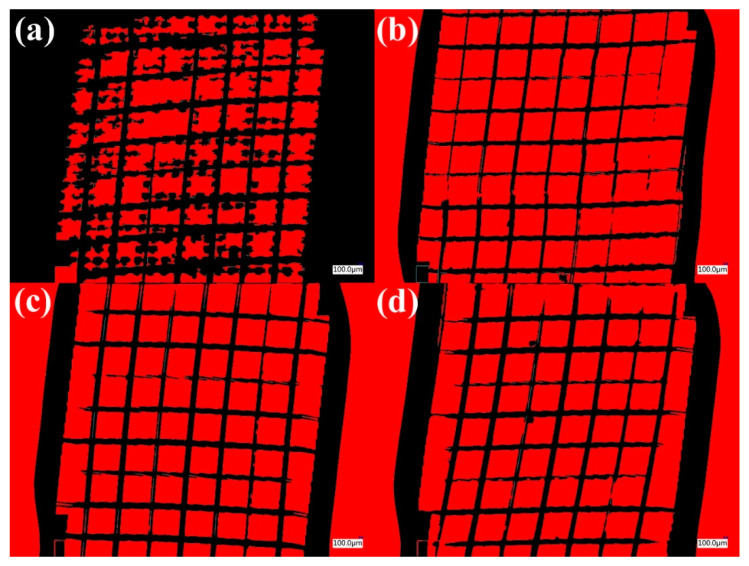
Quantitative analysis of the area detached for coatings using the adhesive tape method and digital microscopy: (**a**) untreated silver coating, (**b**) silver coating with flame treatment, (**c**) untreated WS_2_ coating, and (**d**) WS_2_ coating with flame treatment.

Figure 9 shows a proposed method for measuring the detached area using a quantitative analysis of the detached area by optical digital microscopy [48]. In Figure 9a, the surface without heat treatment had a larger detached area due to poor adhesion to the substrate (70.6% detached area) compared to Figure 9b, the surface with the flame treatment (59.99% detached area). According to ASTM D 3359-23 [45], it could be classified as 1B with >65% detachment, and with the flame treatment it could be classified as 5B [48].

Figure 9c,d compare the WS_2_ surfaces, showing no significant effect from the flame treatment on the coating. According to ASTM D 3359-23 [45], they could be classified as 5B with 0% detachment. Therefore, in the case of WS_2_, the treatment could be dispensed. According to the regulations (ASTM D-3359-23 [45]), a detachment of less than 5% could be considered, which could be classified as 4B.

### 3.3. XPS Analysis of Silver Metallic Film

**Figure 10:**
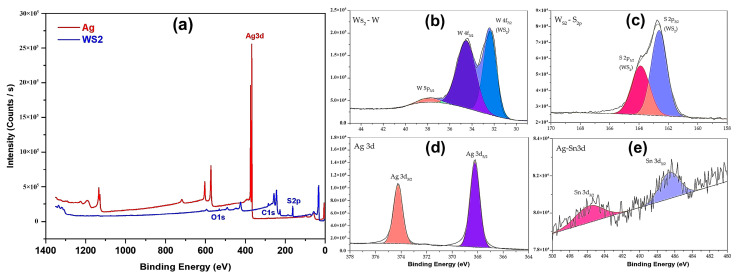
XPS spectra of Ag and WS_2_ reflective coatings. (**a**) Survey, (**b**) W4f and W5p of WS_2_, (**c**) S2p of WS_2_, (**d**) Ag3d of Ag, and (**e**) Sn3d of surface activator.

Figure 10 shows the results of the XPS analyses for Ag and WS_2_ reflective surface samples. Figure 10b,c show the W4f/W5p and S2p of WS_2_, respectively. The high-resolution XPS spectrum of Ag3d_3/2_ and Ag3d_5/2_ was obtained (Figure 10d). Taking as a reference the main peak of metallic silver (368.2 eV), Ag 3d_5/2_, located at 368.2 eV, corresponds to the binding energy of metallic silver [55]. Particularly relevant was a perceptible signal for the Sn used as a surface activator on PC for subsequently obtaining the Ag coating (Figure 10e).

## 4. Conclusions

Metallic electroless silver deposition was accomplished on polycarbonate substrates using the dynamic chemical plating (DCP) technique. The thin films were nanometers in thickness and produced over an area of about 1.5 m^2^ for individual mirror pieces for concentrated solar power facilities.

- DCP is a suitable process for depositing metal layers of Ag on non-conductive polycarbonate surfaces, using double-nozzle spray guns at room temperature. The layers obtained were deposited homogeneously on the substrate and had a uniform thickness and excellent adhesion properties as the deposits obtained on the polycarbonate were uniform. Therefore, DCP is a feasible method that does not require plating tanks, racks, drums, or external electric currents.
- The surface treatment by flame changed the wettability of the surface as the static contact angle with the water decreased. Generally, adequate adhesion was found between the coating and the substrate, quantified accordingly with ASTM D 3359-23 40, obtained from the shear test.
- Analysis of the chemical composition of the DCP deposit on the PC surface showed metallic silver.
- Most solar mirrors used in commercial concentrating solar plants are made with a thin reflective layer of silver (<200 nm) between glass and paints to ensure high reflectance across the solar spectrum, with high durability for decades. In this work, the polycarbonate/Ag system showed an excellent reflectivity value of >90%, comparable to commercial rigid reflectors used in concentrated solar power. The flame treatment improved this reflectivity, which was attributed to the elimination of residues of silver compounds that could be photoactive, darken the surface, or even cause different types of dark spots.
- A specular reflective coating type based on WS_2_ lamellar powder was achieved using easy, dry, and one-step mechanical polishing. Results showed reflectance higher than 90% for the visible region and >99% for the NIR region. Also, it showed adequate adhesion with and without flame treatment, which caused a slight reflectance reduction attributed to lamellar orientation on the surface.
- The combination of a silver coating on the back side of the PC and WS_2_ on the front reduced reflectance, as was expected. However, the change on the external surface could be more extensively studied, as desirable properties can be foreseen. Alternatively, the silver and WS_2_ specular surfaces could also be used separately.
- This work, for the first time, proposes protecting–reflecting on both sides of mirrors and a solution to PC surface vulnerability to weathering and scratching. Even more, using mechanical polishing, WS_2_ is used for reflection and surface protection and can be applied to polymers (PC) and metals (aluminum). The PC pieces were the industrially available substrates that resisted pretreatment with a flame for the silver electroless process.

## Figures and Tables

**Table 1:** Parameters used for flame surface treatment.

Pressure (bar)	Power (kW)	Gas Flow Rate (kg/h)	Power Source
1.5–3.5	45–76	2.9–5.2	Natural gas

**Table 2:** Different thicknesses for each treatment done.

Layers Plating	Thickness (nm)
8	45
16	100
24	150
40	200
48	245

**Table 3:** Scotch test results.

Number of Layers Added to the Substrate	% Area Removed	Classification
8 layers	<5%	4B
16 layers	15–30%	2B
24 layers	35–65%	1B
40 layers	35–65%	1B
48 layers	>65%	0B

## Data Availability

The original contributions presented in the study are included in the article; further inquiries can be directed to the corresponding authors.
